# A standardized production pipeline for high profile targets from *Mycobacterium tuberculosis*

**DOI:** 10.1002/prca.201600033

**Published:** 2016-08-03

**Authors:** Morlin C. Milewski, Tobias Broger, Joanna Kirkpatrick, Angela Filomena, Dana Komadina, Nicole Schneiderhan‐Marra, Matthias Wilmanns, Annabel H. A. Parret

**Affiliations:** ^1^ European Molecular Biology Laboratory (EMBL)Hamburg UnitHamburgGermany; ^2^ Foundation for Innovative New Diagnostics (FIND)GenevaSwitzerland; ^3^ European Molecular Biology Laboratory (EMBL)Proteomics Core FacilityHeidelbergGermany; ^4^ Natural and Medical Sciences InstituteUniversity of TübingenReutlingenGermany; ^5^ University of Hamburg Clinical Center Hamburg‐EppendorfHamburgGermany

**Keywords:** Antigen, *Mycobacterium*, Multiplex immunoassay, Recombinant protein production

## Abstract

**Purpose:**

Tuberculosis is still a major threat to global health. New tools and strategies to produce disease‐related proteins are quintessential for the development of novel vaccines and diagnostic markers.

**Experimental design:**

To obtain recombinant proteins from *Mycobacterium tuberculosis* (Mtb) for use in clinical applications, a standardized procedure was developed that includes subcloning, protein expression in *Mycobacterium smegmatis* and protein purification using chromatography. The potential for the different protein targets to serve as diagnostic markers for tuberculosis was established using multiplex immunoassays.

**Results:**

Twelve soluble proteins from Mtb, including one protein complex, were purified to near‐homogeneity following recombinant expression in *M. smegmatis*. Protein purity was assessed both by size exclusion chromatography and MS. Multiplex serological testing of the final protein preparations showed that all but one protein displayed a clear antibody response in serum samples from 278 tuberculosis patients.

**Conclusion and clinical relevance:**

The established workflow comprises a simple, cost‐effective, and scalable pipeline for production of soluble proteins from Mtb and can be used to prioritize immunogenic proteins suitable for use as diagnostic markers.

**Abbreviations**
HIC: hydrophobic interaction chromatography
IEX: ion exchange chromatography
IgG: immunoglobulin G
Mtb: *Mycobacterium tuberculosis*
PE: protein extraction
TB: tuberculosis

## 1 Introduction

Tuberculosis (TB) is far from being a disease of the past. Each year 1.5 million die as a result of TB, a highly infectious and complex illness caused by the airborne bacterium *Mycobacterium tuberculosis* (Mtb) 1. The WHO End TB Strategy (http://www.who.int/tb/post2015_strategy/en/) aims to end the global TB epidemic, with targets to reduce TB deaths by 95% and incidence rate by 90% between 2015 and 2035. Moving forward to the 2035 targets requires the ensured availability of new tools, including (i) affordable and highly sensitive diagnostic tests for all forms of TB that can be implemented at the point of care, (ii) a vaccine that protects those of all ages who are not yet infected–preferably one that can also prevent people with latent TB from progressing to active disease and (iii) highly effective, shorter drug regimens, including regimens for TB infection caused by drug‐resistant TB. The development of vaccines, diagnostics, and drugs depends upon a fundamental knowledge of biochemical pathways and intracellular processes critical for Mtb pathogenesis 2. In recent years, significant progress has been made by the use of MS‐based proteomics studies (reviewed in 3, 4). However, the majority of research efforts aims at characterizing individual Mtb virulence factors and their interaction with host targets by conventional genetic, biochemical, and biophysical methods (reviewed in 5). Protein expression and purification are invariably key features of these studies.

**Clinical Relevance**

In 2014, tuberculosis (TB) killed 1.5 million people and now ranks alongside HIV as a leading cause of death worldwide. The causative agent of TB is the airborne bacterium *Mycobacterium tuberculosis* (Mtb), which mainly attacks the lungs and is easily transmittable through inhalation of aerosol droplets. To open new opportunities for prevention and innovative therapies, it is essential to gain a better mechanistic understanding of the underlying molecular processes both within the pathogen and its interactions with the human host during infection. Progress toward understanding the structure and function of Mtb proteins has, however, been hampered by a scarcity of easily applicable protocols and tools for producing sufficient amounts of recombinant protein for clinical applications, such as vaccine development and biomarker identification. Here, we describe an effective expression and standardized expression workflow to rapidly assess whether functional Mtb proteins can be produced via recombinant expression in nonpathogenic *Mycobacterium smegmatis*. Our results show that *M. smegmatis* is a valuable host for the efficient production of immunogenic proteins from Mtb, which may advance the development of tools for better diagnosis, prevention, and treatment of TB.

It is generally accepted that the bottleneck in recombinant protein production is obtaining sufficient amounts of soluble and functionally competent protein for downstream studies. While *Escherichia coli* is still the default expression host for recombinant protein production, it lacks species‐specific chaperones for correct folding, which could drive recombinant proteins into insoluble inclusion bodies 6. Insolubility and misfolding can also result from the mismatch between the codon usage of *E. coli* and the protein of interest 7. In case of proteins from mycobacterial species, including Mtb, typically only one third of the proteins expressed in *E. coli* are produced as soluble protein 8, 9, 10. In this work, we therefore exploited the fast‐growing saprophytic bacterium *Mycobacterium smegmatis* as an expression host, which provides a more suitable background for the production of proteins that resemble the native protein 11, 12. Our comprehensive protocols use an optimized expression host and optimized expression vectors for inducible expression in *M. smegmatis*, in amounts and quality amenable for downstream applications such as serodiagnostic tests or functional characterization. The pipeline has been evaluated for the expression, solubility, and amenability for purification of 25 constructs derived from 18 Mtb targets with diverse functional and biochemical properties.

## 2 Materials and methods

### 2.1 Protein expression in *M. smegmatis*

Expression vectors, bacterial strains and growth conditions used in this study are described in the Supporting Information Table 1. *M. smegmatis* starter cultures were cultivated from freshly streaked colonies or glycerol stocks for 3 days at 37°C. For small‐scale and large‐scale expression studies, 1% of a starter culture was used to inoculate 100 mL or 1 L 7H9 expression medium, respectively. When cultures had reached an optical density of ≈2.5 measured at 600 nm, protein expression was induced with 34 mM acetamide (Sigma‐Aldrich, Germany) and cultivation was executed for an additional 16–24 h. Cells were harvested by centrifugation at 3220 × *g* or 7550 × *g* for small‐scale and large‐scale purification, respectively. Pellets were flash‐frozen in liquid N_2_ and stored at –80°C until further use.

### 2.2 Protein purification

In brief, crude extracts were obtained by centrifugation for 45 min at 4**°**C (40 000 × g) after sonication of the cells in 20–25 mL protein extraction (PE) buffer (300 mM NaCl, 50 mM HEPES pH 8.0, 20 mM imidazole) as described previously 13. The supernatant was filtered through a 0.45 μm filter and applied onto a Poly‐Prep column (Bio‐Rad, Germany) containing 200 μL (for 50 mL purification) or 1 mL (for 1 L purification) Ni‐NTA agarose (Qiagen, Germany) preequilibrated with PE buffer. Protein contaminants were removed by multiple washing steps using PE buffer. His‐tagged proteins were eluted with 3–5 column volumes PE buffer containing 500 mM imidazole. All purification fractions were collected and analyzed on NuPAGE Novex 4–12% Bis‐Tris gels (Life Technologies GmbH, Germany). SDS‐PAGE gels were stained with Instant Blue gel stain (Expedeon, Cambridgeshire, UK) that has a sensitivity of 5–25 ng protein/band. Protein size was verified by comparison with the Rotimark 10–150 protein marker (Carl Roth, Germany). For final protein preparations, recombinant protein was dialyzed into SEC buffer (50 mM HEPES pH 8.0, 100 mM NaCl) and passed through a SEC column (HiLoad 16/60 Superdex 75 or 200, GE Healthcare Life Sciences, Germany) using SEC buffer as running buffer. SEC columns were calibrated using a mixture of four proteins, thyroglobulin (670 kDa), γ‐globulin (158 kDa), ovalbumin (44 kDa), and myoglobin (17 kDa) (Gel Filtration Standards mixture, Bio‐Rad, Richmond, CA, USA). Protein purity was verified by SDS‐PAGE and fractions containing pure protein were pooled, flash‐frozen in liquid N_2_ and stored at –80°C. When additional chromatography steps were necessary in order to achieve 90% protein purity, protein samples were dialyzed or desalted using PD‐10 columns (GE Healthcare Life Sciences) into ion exchange (IEX) buffer (50 mM HEPES pH 8.0, 100 mM NaCl) or hydrophobic interaction chromatography (HIC) starting buffer (50 mM HEPES pH 8.0, 1 M (NH_4_)_2_SO_4_). For IEX, proteins were applied to a MonoQ 5/50 GL column (GE Healthcare Life Sciences) and eluted with a linear gradient of 10 column volumes from 100 mM to 500 mM NaCl. Fractions containing the target protein were identified by SDS‐PAGE, pooled, and protein buffer was exchanged to SEC buffer or HIC buffer depending on the necessary subsequent step. Proteins to be further purified by HIC were applied to a 1 mL Phenyl Sepharose HP column (GE Healthcare Life Sciences) and eluted with a linear gradient of 100–0% (NH_4_)_2_SO_4_. Additional details with respect to protein purification are described in the supplementary information. Protein sequences are given in Supporting Information Fig. 1.

### 2.3 Mass spectrometry

Intact protein molecular weight analysis was performed using LC‐MS on a Q‐TOF mass spectrometer (Waters GmbH, Germany). Protein samples in solution were acidified and subjected to C_4_ reverse phase chromatography before electrospray ionization in positive ion mode. Raw data charge state series spectra were deconvoluted to neutral molecular weight using a maximum entropy algorithm. Protein digestion was carried out either in‐gel or in‐solution with different enzymes and the resulting peptides were analyzed by nano‐LC‐MS/MS on an Orbitrap mass spectrometer (Fisher Scientific, Germany). Data processing was performed using Mascot (Matrix Science Ltd., UK) as the search engine. Detailed protocols are provided as supplementary information. The MS proteomics data have been deposited to the ProteomeXchange Consortium via the PRIDE 14 partner repository with the dataset identifier PXD004133.

### 2.4 Multiplex immunoassay

The functionality of the proteins was tested by measuring immunoglobulin G (IgG) antibody response in serum samples from 445 well‐characterized TB suspects from Vietnam and Peru (167 Non‐TB and 278 TB) as described previously 15. Specimens were provided by the Foundation of Innovative New Diagnostics (FIND) and tested at the Natural and Medical Sciences Institute (NMI, University of Tübingen, Reutlingen, Germany). In brief, the purified proteins were covalently coupled to color‐coded beads (MagPlex Microspheres, Luminex Corp, Austin, USA). The bead mixture was incubated with patient sera and protein‐bound human antibodies were detected with PE‐labeled anti‐human IgG on a Luminex FlexMAP3D (Luminex Corp). Median fluorescence intensity was calculated for every sample based on >60 measured beads per bead sort and *z*‐scores were used to illustrate antibody responses per patient in relationship to the mean of all patients. Receiver operating characteristic (ROC) curves were used to illustrate the discriminating utility of the best performing protein and Mann–Whitney *U*‐test was used to determine whether the antibody reactivity in the two populations (TB and Non‐TB) is significantly different.

## 3 Results and discussion

Our overall goal was to develop a standard purification protocol with a minimum number of steps facilitating the production of milligram‐amounts of proteins of at least 90% purity for subsequent testing of antibody binding in serum samples from TB suspects. Purity guidelines were guided by typical sample requirements for downstream applications, such as structural analysis and serodiagnostic assays.

### 3.1 Target selection, construct design, and cloning of *M. tuberculosis* targets proteins

A panel of 18 diverse Mtb proteins previously associated with antibody reactivity in sera from TB patients 16 were selected to validate our procedure (Table 1). Targets were distributed across four functional groups, namely lipid metabolism; cell wall and cell processes; intermediary metabolism and respiration or virulence, detoxification, adaptation. Most proteins were either associated with membrane preparations 17 and/or culture supernatant filtrate 18, 19, 20, 21. At least eight proteins (PstS1, LprG, Acr, EspC, EspA, Ag85a, EsxA, EsxB) represented known virulence factors of the Mtb complex (reviewed in 5), several of which play an essential role in Mtb pathogenicity. Molecular weights of selected target proteins ranged between 7.7 and 39.9 kDa with a p*I* varying from 4.5 to 7.8. The heterodimeric complex of EsxB and EsxA (further referred to as EsxBA) was included as an internal reference as it was shown previously to be efficiently produced in *M. smegmatis* 22.

**Table 1:** *M. tuberculosis* H37Rv proteins expressed in *M. smegmatis groEL1ΔC*

Gene number	Protein name^a^	Uniprot entry^b^	Protein identity	Functional group^c^	Subcellular location^d^	Molecular mass (kDa)^e^	Isoelectric point^f^
Rv0632c	EchA3	P96907	Enoyl‐CoA hydratase	1	n.a.	24.4	5.52
Rv0934	PstS1	P9WGU1	Phosphate‐specific transport substrate‐binding protein‐1	2	M; S^18^	38.2	5.14
Rv1411c	LprG	P9WK45	Lipoarabinomannan carrier protein	2	M; S^19^	24.5	7.78
Rv1837c	GlcB	P9WK17	Malate synthase G	3	C; S^18^	80.4	5.03
Rv1860	Apa (Mpt32)	P9WIR7	Alanine and proline‐rich secreted protein	2	S^18^	32.7	4.93
Rv1886c	Ag85b (FbpB)	P9WQP1	Diacylglycerol acyltransferase/mycolyltransferase	1	S^18^	34.6	5.62
Rv1980c	Mpt64	P9WIN9	Immunogenic protein	2	S^18^	24.9	4.84
Rv2031c	Acr (HspX)	P9WMK1	Alpha‐crystallin	0	C; M	16.2	5.00
Rv2654c	Antitoxin Rv2654	P9WJ11	Antitoxin component of a toxin‐antitoxin module (Rv2654c‐Rv2653c)	0^g^	n.a.	7.7	5.04
Rv2873	Mpt83	P9WNF3	Immunogenic cell surface lipoprotein	2	M; S^18^	22.1	4.86
Rv3615c	EspC	P9WJD7	ESX‐1 secretion‐associated protein	2	M; S	10.8	5.10
Rv3616c	EspA	P9WJE1	ESX‐1 secretion‐associated protein	2	M; S	39.9	5.19
Rv3804c	Ag85a (FbpA)	P9WQP3	Diacylglycerol acyltransferase/mycolyltransferase	1	C; M; S^18^	35.7	6.08
Rv3841	BfrB	P9WNE5	Ferritin	3	C; M; S^18^	20.4	4.73
Rv3864	EspE	P9WJD3	ESX‐1 secretion‐associated protein	2	M	42.1	4.72
Rv3874	EsxB	P9WNK5	ESAT‐6‐like protein CFP‐10	2	S^18^	9.9	4.59
Rv3875	EsxA	P9WNK7	6 kDa early secretory antigenic target ESAT‐6	2	S^18^	10.8	4.48
Rv3881c	EspB	P9WJD9	ESX‐1 secretion‐associated protein	2	S^18^	47.6	4.75

a) Commonly used alternative names are indicated in brackets.

b) Protein accession number in UniProt database (http://www.uniprot.org/) version July 2016.

c) Explanation of functional group: (0) virulence, detoxification, adaptation, (1) lipid metabolism, (2) cell wall and cell processes, (3) intermediary metabolism and respiration. Functional group codes are taken from the web server (http://genolist.pasteur.fr/TubercuList/).

d) The subcellular location of each target as indicated in the UniProt database. For certain targets additional data on subcellular localization was retrieved from selected publications.

e) Molecular weights of apo‐proteins without protein tags as reported in the Uniprot database.

f) Isoelectric points were calculated using the Protparam tool (http://web.expasy.org/protparam/).

g) This protein is wrongly functionally categorized as “insertion sequences and phages.” According to Ramage et al., protein Rv2654 is the antitoxin component of a toxin‐antitoxin module 34 and has therefore been categorized in this study as “virulence, detoxification, and adaptation protein.”

For the well‐established and most commonly used *E. coli* expression platform, an extensive catalog of molecular tools is available, which greatly simplify recombinant protein production 7. In contrast, expression vectors and protocols geared toward high‐yield expression in mycobacteria are rather limited (reviewed in 9). In this study, we used in‐house generated inducible expression vectors, pMyNT and pMyC 22. These *E. coli*/mycobacterial shuttle vectors contain an acetamidase promoter 23, 24, 25, a hygromycin resistance gene and an N‐ or C‐terminal hexahistidine‐tag in case of pMyNT or pMyC, respectively. Polyhistidine‐tags substantially facilitate protein purification, while rarely adversely affecting protein structure or function 26. Using IMAC, polyhistidine‐tagged proteins can be enriched 100‐fold in a single step resulting in high yields of up to 95% pure protein 27. In case the affinity tag would influence protein solubility or function, pMyNT contains a recognition site for tobacco etch virus protease allowing for its efficient removal from the recombinant protein by proteolytic cleavage.

One to three constructs were designed per target gene including the full‐length gene sequence. For targets reported to be secreted or associated with the Type VII secretion system we prepared additional constructs to express the apo‐proteins with a C‐terminal hexahistidine‐tag. For six targets with experimentally validated N‐terminal signal sequences 28, truncated constructs were created in order to produce mature protein.

### 3.2 Expression screening and solubility assessment

**Figure 1:**
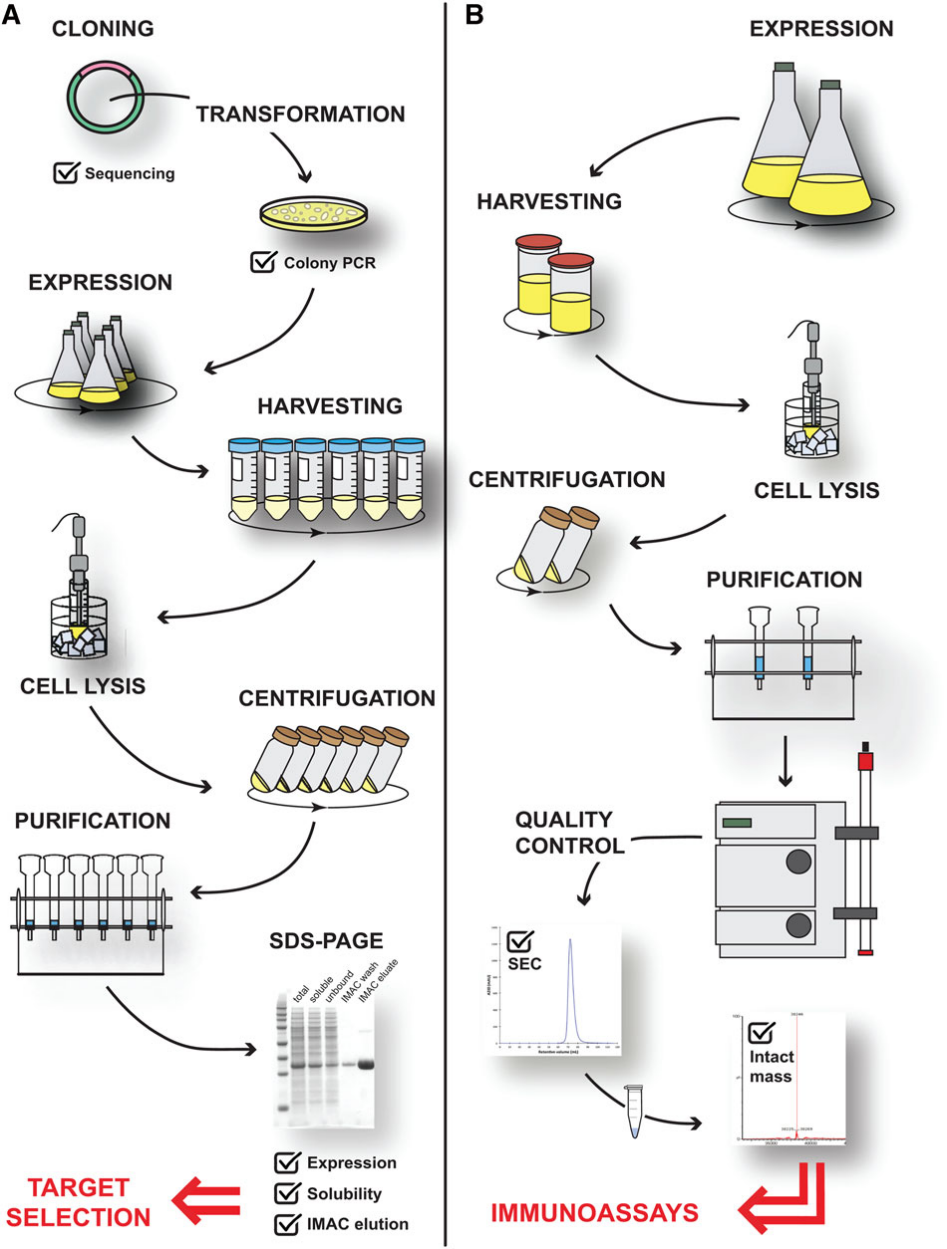
Schematic overview of established workflow used in this study to produce recombinant proteins from *M. tuberculosis* in *M. smegmatis*. Essential checkpoints throughout the workflow are indicated. (A) Workflow for small‐scale expression and solubility assessment from 100 mL *M. smegmatis* cultures. (B) Scale‐up of protein purification for promising Mtb targets including a polishing step using SEC and protein identification by MS analysis.

**Figure 2:**
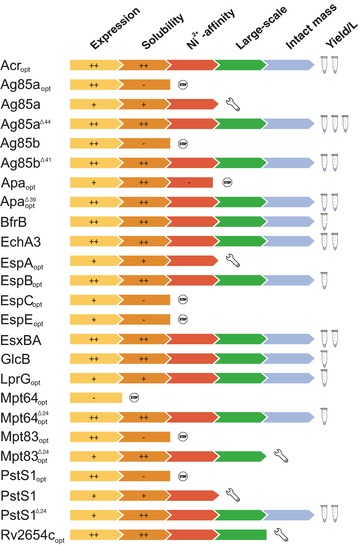
A schematic representation of the various steps leading toward purification of Mtb targets from *M. smegmatis* starting from expression constructs. Purification yields are shown by one, two, or three “tube” symbols denoting a protein yield of <1 mg/mL, between 1 and 5 and above 5 mg protein per liter expression culture, respectively. Exact protein yields are given in Supporting Information Table 2. “Stop” symbols indicate constructs not withheld due to insufficient protein yield or purity. Five constructs were promising in initial large‐scale expression and purification, although these would require optimization of purification protocols in order to obtain sufficient protein for further studies (indicated by a “wrench” symbol).

Following sequence verification, selected constructs were transformed to *M. smegmatis groEL1ΔC* by electroporation (Fig. 1). This modified expression strain was generated in our laboratory and is ideally suited for expression of polyhistidine‐tagged proteins due to a deletion of the histidine‐rich C‐terminal tail of the common contaminant GroEL1, thus consequently increasing the speed and efficiency of protein purification using IMAC 13. Initial expression experiments were performed in 100 mL cultures in rich growth medium, where expression was induced for 24 h. Cells from 50 mL cultures were lysed in a standardized lysis buffer and soluble protein was recovered after high‐speed centrifugation. The lysis step is the only step in our workflow where targets cannot be handled simultaneously. Soluble proteins were further mixed with Ni‐NTA agarose beads to capture polyhistidine‐tagged proteins in batch‐binding mode using reusable plastic columns. The IMAC step was designed in such a way that the complete experiment could be carried out on a work bench without the need of specialized equipment. In this way, lysates from six constructs could be easily processed in parallel. Binding, washing, and elution of tagged proteins were achieved by gravity flow purification. Whole cell lysate and protein preparations were resolved by SDS‐PAGE and the expression level, solubility, and IMAC recovery of target proteins was assessed by inspection of Coomassie‐Blue‐stained SDS‐polyacrylamide gels. The levels of total expression for each target were scored visually using a standardized criterion; no or very low expression (no visible bands); medium (adequately sized band); high (very intense band). Expression of 24 out of 25 constructs was detectable by Coomassie staining (Fig. 2). Seventeen constructs, corresponding to 15 unique targets, yielded highly expressed protein, and nine constructs expressed moderately. Given that our aim was to select promising constructs for high yield protein production from cellular extracts, low expressing constructs beyond our limits of detection were not considered further.

All proteins ran at the expected molecular weight on SDS‐PAGE, with the exception of the proline‐rich Apa protein (Supporting Information Fig. 3). For both constructs of Apa, an aberrant migratory behavior on SDS‐PAGE was observed. However, proteins with high‐proline content are notorious for running slower than their actual molecular weight during SDS‐PAGE 29. Alternatively, the presence of carbohydrate appendages could also result in a higher apparent molecular weight. However, this was ruled out by subsequent MS characterization (further details see below).

The solubility of each construct was further evaluated by comparing equal amounts of total protein before and after removal of insoluble material. Protein constructs were categorized as being either soluble (corresponding amounts of protein in total versus soluble protein fractions), insoluble (protein only detected in total protein fraction) or partly soluble (Fig. 2). Overall, constructs expressing well also yielded highly soluble protein. EspC and EspE were not pursued for further analysis due to low expression and solubility combined. Expression of another four full‐length constructs (Ag85a_opt_, Ag85b, Mpt83_opt_, PstS1_opt_) was highly efficient, but did not yield soluble protein using standard buffer conditions for cell lysis. Further screening and optimization of cell lysis conditions would be necessary in order to rescue these particular targets. For our study, we rather opted to purify the corresponding proteins without a signal peptide.

Finally, the recovery after IMAC was evaluated by analyzing the proteins that eluted from the Ni‐NTA resin at high imidazole concentrations. In this study, all targets that were shown to be soluble under the conditions evaluated, could also purified by IMAC, with the exceptions of Apa_opt_.

### 3.3 Large‐scale purification of selected targets and quality control

On the basis of the expression levels, solubility, and purification criteria described above, 14 constructs were selected for upscaling, corresponding to 15 unique Mtb targets. Figure 2 summarizes the purification pipeline for each target as well as the obtained yield per liter of expression culture. For each target one construct was selected, exhibiting the most promising protein yield in large‐scale purification. Three ESX‐1–associated proteins (EspA, EspC, EspE) were not deemed suitable for upscaling as part of this investigation. In order to purify large quantities of these proteins, further optimization of expression and purification protocols would be indispensable.

To this end, recombinant proteins were produced in 1–2 L bacterial cultures and purified from contaminants by upscaling the methods employed in the small‐scale screening using identical protein extraction and IMAC buffers (Fig. 1B). In order to achieve the defined sample requirements, in some cases an IEX or HIC step was included in order to remove protein contaminants (Supporting Information Table 2). Final polishing was achieved by SEC, which allows the removal of unwanted protein aggregates (Supporting Information Fig. 2). This technique is considered to be a crucial quality control method in the field of protein production 30, 31. SEC analysis established conformational homogeneity of all produced proteins. Comparison of the SEC elution profiles with the elution profile of a set of known molecular mass standards, allows a rough estimation of the oligomeric state of the obtained proteins. Four proteins eluted at the expected retention volume corresponding to monomeric protein (EsxBA, GlcB, Mpt64_opt_, PstS1^Δ24^), while both Ag85a^Δ44^ and Ag85b^Δ41^ migrated somewhat slower than expected in case of a globular protein of 34 and 33 kDa, respectively. This phenomenon could be explained by retention of the proteins due to nonspecific binding to the SEC column. Six proteins (Acr_opt_, Apa_opt_, BfrB, EchA3, EspB_opt_, LprG_opt_) eluted from the SEC column earlier than globular proteins of corresponding molecular weights. This could be due to an extended protein structure resulting in a larger hydrodynamic radius and hence faster elution rate through the SEC column. Alternatively, it could indicate that the protein exists as stable oligomer in solution. The latter option clearly applies to BfrB. The derived molecular mass of BfrB (± 460 kDa) is consistent with the observation that this protein exists as a 24‐mer in solution 32.

It should be noted that techniques such as dynamic light scattering or multiangle static light scattering combined with SEC would provide a far more accurate determination of the molecular weight of proteins. However, such an advanced biophysical analysis was beyond the scope of this study.

### 3.4 Mass spectrometry

To verify the identity and purity of the produced proteins, we performed intact mass measurements by LC‐MS (Supporting Information Table 3). Intact protein masses matched the theoretical molecular weights for all proteins within the mass accuracy limits of the instrument (1–2 Da). No evidence for PTMs was found, e.g. phosphorylation or glycosylation. However, the intact mass of GlcB appeared 162 Da higher than its theoretical mass. A more in‐depth MS analysis would be necessary to determine the underlying reason for this mass difference. Two proteins were not amenable to intact mass determination (EsxA, LprG_opt_), but protein identity was confirmed by follow‐up peptide mass fingerprinting analysis (Supporting Information Table 4). However, in case of LprG_opt_, it should be noted that the MS analysis indicated that this protein preparation was quite significantly contaminated with endogenous *M. smegmatis* proteins, as can also be seen in the final SDS‐PAGE analysis (Supporting Information Fig. 3). In case of EspB_opt_, we noticed the presence of two distinct protein species (denoted as peak A and peak B) that eluted at slightly different elution volumes during IEX chromatography. Both peaks were analyzed separately by SEC (Supporting Information Fig. 2), intact mass determination (Supporting Information Table 3) and peptide mass fingerprinting (Supporting Information Table 4), but no noticeable differences could be observed. EspB_opt__peak B was used in further experiments due to the higher yield that was obtained for this species.

### 3.5 Antibody detection in serum samples

**Figure 3:**
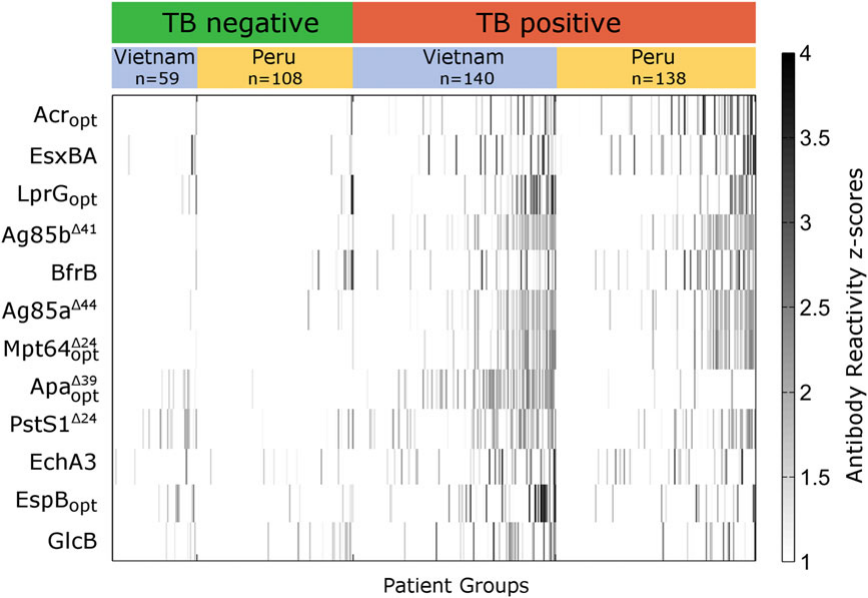
Antibody response profiles of 445 patient samples (167 Non‐TB and 278 TB) show significantly higher reactivity in 11 of the 12 targets (all expect GlcB). Gray scaled *z*‐scores (standard deviation above mean) indicate relative antibody levels. One standard deviation above the mean signal was used as a cut‐off leading to white color for samples with lower antibody reactivity.

We investigated the immune response against proteins produced in our workflow in TB patients using a multiplex immunoassay. To compare assay performance to WHO targets (http://www.who.int/tb/publications/tpp_report/en/) sensitivity was calculated at a preset 95% specificity (targeting a TB detection test) and specificity was calculated at a preset sensitivity of 90% (targeting a TB triage test). A significantly higher antibody response in the TB compared to the non‐TB group was shown for 11 out of 12 (92%) proteins, confirming binding of most proteins to human IgG (Table 2). Ag85b reached the highest sensitivity (36%) among all proteins at a specificity of 95%. Figure 3 shows antibody profiles against all proteins in this study (most significant proteins on top) for all 445 patients to visually illustrate the significantly higher antibody response in the TB group for the 11 discriminatory proteins. Acr_opt_ displayed the most elevated antibody response. For GlcB, the IgG response was not significantly different in the TB versus the non‐TB group, suggesting that either the purification did not result in a functional product or that there is no significant antibody response toward GlcB in the course of TB infection. Additional functional and biochemical analyses would be required to investigate this in more detail.

**Table 2:** Significance and diagnostic performance of Mtb proteins in antibody detection assays

Protein	Mann–Whitney *U* test (*p*‐value)	ROC‐AUCa,, b	Detection test sensitivity (at 95% specificity)	Triage test specificity (at 90% sensitivity)
WHO target performance	c	> 0.90	> 0.65	> 0.70
Acr_opt_	6.15 × ^–10^	0.68	0.26	0.17
EsxBA	1.88 × ^–9^	0.67	0.20	0.22
LprG_opt_	5.20 × ^–9^	0.67	0.18	0.24
Ag85b^Δ41^	6.81 × ^–9^	0.66	0.36	0.18
BfrB	7.37 × ^–9^	0.66	0.16	0.21
Ag85a^Δ44^	1.82 × ^–8^	0.66	0.32	0.16
Mpt64 opt Δ24	1.81 × ^–7^	0.65	0.34	0.17
Apa opt Δ39	4.26 × ^–7^	0.64	0.23	0.20
PstS1^Δ24^	5.35 × ^–7^	0.64	0.12	0.21
EchA3	4.32 × ^–5^	0.62	0.17	0.14
EspB_opt_	4.58 × ^–5^	0.62	0.12	0.16
GlcB	3.24 × ^–1^ d	0.53	0.10	0.14

a) The diagnostic performance of two hypothetical TB tests (detection test and triage test) as they are targeted by the WHO (http://www.who.int/tb/publications/tpp_report/en/) were calculated as follows: sensitivity values of a detection test having a high specificity of 95% and specificity of a triage test having a high diagnostic sensitivity of 90%, respectively.

b) Area under the receiver operating characteristic curve.

c) Not applicable.

d) Not significant at a significance level of > 0.05.

## 4 Concluding remarks

We presented here an efficient workflow for screening Mtb targets for production as recombinant proteins in the related fast‐growing expression host *M. smegmatis*. As the proteins were of particular functional interest, considerable effort was made to ensure targets moved through the production pipeline with the aim to obtain as many targets as possible with at least 90% purity. Twelve targets were purified successfully from 1–2 L bacterial cultures following a standardized workflow where possible. Using this approach, we were able to rapidly select the optimal expression constructs for selected Mtb targets. All proteins were delivered to the Foundation for Innovative New Diagnostics (FIND) to assess their suitability as diagnostic markers for TB. Initial data showed increased antibody binding in the TB versus the non‐TB group, but relatively low diagnostic performance. In follow‐up experiments a comprehensive analysis of host‐dependent protein modifications versus immunological differences (e.g. a direct comparison with native proteins purified from Mtb or *E. coli*) will shed light on this issue. In this respect, it would be very valuable to extend the biophysical and biochemical characterization of the most promising protein targets in order to identify possible cooccurring protein species that could explain the relatively low diagnostic performance 33. Furthermore, the performance and the feasibility of measuring IgG antibody response in combination with other markers await further efforts. Finally, our study provides a set of standardized protocols for rapid screening of Mtb proteins amenable for structural analysis.

*The authors have declared no conflict of interest*.

## Supporting information

As a service to our authors and readers, this journal provides supporting information supplied by the authors. Such materials are peer reviewed and may be re‐organized for online delivery, but are not copy‐edited or typeset. Technical support issues arising from supporting information (other than missing files) should be addressed to the authors.

**Table S1** Plasmids and oligonucleotides used for cloning protein encoding genes and for recombinant protein expression.
**Table S2** Purification strategy and protein yields after large scale expression of selected proteins
**Table S3** Intact mass measurements by LC‐MS
**Table S4** Protein identification analysis of the protein digests (LC‐MS/MS) carried out for a subset of proteins
**Figure S1** Protein sequences
