# Supplementary material for: A standardized production pipeline for high profile targets from Mycobacterium tuberculosis
Source: Proteomics Clin Appl. 2016 Aug 3;10(9-10):1049–57. doi: 10.1002/prca.201600033 (PMC5095800; doi:10.1002/prca.201600033)
Supplement: Supplementary file 1 — Table S1 Plasmids and oligonucleotides used for cloning protein encoding genes and for recombinant protein expression. Table S2 Purification strategy and protein yields after large scale expression of selected proteins Table S3 Intact mass measurements by LC‐MS Table S4 Protein identification analysis of the protein digests (LC‐MS/MS) carried out for a subset of proteins Figure S1 Protein sequences [file PRCA-10-1049-s001.docx]

**Supporting information, figures and tables**

**Media, strains and growth conditions.** All cloning was done in *E. coli* strain DH5α-T1^R^ (Life Technologies GmbH, Darmstadt, Germany). *E. coli* was transformed by conventional heat shock transformation [1] and grown in Luria-Bertani (LB) low salt broth or on LB high salt plates (Melford Laboratories Ltd, Ipswich, UK). *M. smegmatis* mc^2^155 *groEL1ΔC* was grown in Middlebrook 7H9 medium (BD Biosciences, Heidelberg, Germany) or on 7H10 agar plates (Sigma-Aldrich, Schnelldorf, Germany) plus 10% albumin–dextrose saline (ADS: 5% (w/v) BSA cold ethanol fraction, pH 5.2, ≥96% (Sigma-Aldrich), 2% (w/v) glucose (Carl Roth GmbH + Co. KG, Karlsruhe, Germany), 342 mM NaCl), 0.05% (v/v) Tween-80 (Carl Roth) and 0.2% (v/v) glycerol (Carl Roth) respectively. For expression of recombinant proteins, Middlebrook 7H9 medium was used without ADS but supplemented with 0.2% (w/v) glucose. All bacterial strains were grown at 37**°**C. Where appropriate, 94 µM hygromycin B (Carl Roth) was added to growth media.

**Generation of expression constructs**

Vectors pMyNT and pMyC were used for over-expression of *Mtb* genes in *M. smegmatis* generating either N-terminally or C-terminally His_6_-tagged proteins, respectively [2]. Both vectors have been fully sequenced and DNA has been deposited at Addgene (http://www.addgene.org/). Expression constructs were generated by conventional restriction enzyme cloning. Vectors were linearized by restriction enzyme digestion with *Nco*I/*Hin*dIII or *Nco*I/*Bam*HI as indicated in Table S1 followed by dephosphorylation using Antarctic phosphatase (New England Biolabs GmbH, Frankfurt, Germany). *M. tuberculosis* genes were PCR-amplified from *M. tuberculosis* H37Rv genomic DNA or from synthetic DNA constructs codon-optimized for expression in *M. smegmatis* mc^2^155 (designed and provided by Genscript (Genscript, Piscataway, USA) as pUC57-Kan clones). PCR amplification of the target region was performed with Q5® High-Fidelity DNA Polymerase (New England Biolabs) using primers listed in Table S1. Alternatively, Mtb synthetic genes were obtained directly from the pUC57-Kan clones by restriction digest with the appropriate restriction enzyme mixture. For cloning into expression vectors, insert DNA was prepared by restriction digest with *Pci*I/*Bam*HI, *Nco*I/*Bam*HI or *Nco*I/*Hin*dIII. Prior to DNA ligation, we purified all DNA fragments with the Wizard SV Gel and PCR Clean-up System (Promega GmbH, Mannheim, Germany). Ligation mixtures were transformed to *E. coli* DH5α-T1^R^ and transformants were selected on LB plates containing hygromycin. Plasmid DNA was prepared using QIAprep Spin Miniprep kit (Qiagen, Hilden, Germany) and sequence-verified with vector-specific primers (AP-328, 5’-CGCAGTTGTTCTCGCATACC-3’ and pMyNT-rev, 5’- TGGATCTCTCCGGCTTCAC-3’) before transformation to *M. smegmatis.* Electrocompetent *M. smegmatis* were generated and transformed with up to 1 μg plasmid DNA as previously described [3]. Transformants were selected on media supplemented with hygromycin and verified by colony PCR with One*Taq* 2X Master Mix (New England Biolabs) using AP-328 and pMyNT-rev primers according to the supplier’s manual.

**Protein purification**

Generally, target proteins that scored medium to high for total expression and solubility and could be recovered by IMAC, were deemed suitable for scaling up. Prior to all chromatography steps, proteins were filtered with 0.22 μm filter syringes and concentrated to 0.5-5 mg/ml using Spin-X centrifugal concentrators (Corning BV Life Sciences, Amsterdam, Netherlands). Recombinant protein production was quantified spectrophotometrically with A280 nm readings using a theoretical extinction coefficient as calculated by the Protparam tool (http://web.expasy.org/protparam/). For some proteins, some exceptions to the general protocol described in the main text were applied resulting in higher protein yield. Large-scale purification of LprG was achieved using modified extraction (50 mM sodium phosphate pH 8.0, 300 mM NaCl, 20 mM imidazole, 1% protease inhibitor mix), wash (50 mM sodium phosphate pH 8.0, 1 M NaCl, 20 mM imidazole, 10% glycerol) and elution (50 mM sodium phosphate pH 8.0, 300 mM NaCl, 450 mM imidazole) buffers to increase protein purity. The IMAC buffers used for EchA3 purification contained 300mM NaCl rather than 100mM, while for EsxBA 300 mM NaCl was maintained in all purification buffers. For BfrB, 2 mM β-mercaptoethanol was added to all buffers. For antitoxin Rv2654c, 10% glycerol as well as 10 mM MgCl_2_ was added to purification buffers.

**Intact Protein LC-MS**

# Protein samples were acidified using 1% TFA prior to injection onto the Acquity UPLC System (Waters GmbH, Eschborn, Germany). Approximately 3-4 µg of each sample were loaded onto a protein separation column (Waters ACQUITY UPLC Protein BEH C_4_ Column, 300 Å, 1.7 µm, 2.1 mm X 150 mm). The outlet of the analytical column was coupled directly to a Q-Tof Premier mass spectrometer (Waters) using the standard ESI source in positive ion mode. Solvent A was water, 0.1% formic acid and solvent B was acetonitrile, 0.1% formic acid. The samples were loaded in 96% A, 4% B at a constant flow of 0.2 mL/min. The column was held at 4% B for 5 min before ramping to 25% B by 6 min. A linear gradient to 80% B was then applied until 17 min.

For the Q-Tof, a spray voltage of 3.5 kV was applied with a cone voltage of 35 V and extraction cone at 5 V. The desolvation temperature was set at 350 °C, with source temperature 120 °C. Desolvation gas was nitrogen at a flow rate of 600 L/min. Collision energy was set at 5 eV with argon in the collision cell at a pressure of 5.3 e^-3^ mbar. Data was acquired in continuum mode over the mass range 500-3500 *m/z* with a scan time of 0.5 s and an interscan delay of 0.1 s. The instrument was externally calibrated with sodium iodide, against which a reference standard of intact myoglobin was checked immediately prior to sample data acquisition. Spectra from the chromatogram protein peak were then summed and intact mass was calculated using the MaxEnt1 maximum entropy algorithm (Waters) to give the zero charge deconvoluted molecular weight.

**Sample preparation – in solution digestion with trypsin, MALP and WALP**

Protein samples in solution were taken and digested with either trypsin (Promega), WaLP or MaLP (Sigma Aldrich); wild type α-lytic protease (WaLP) and an active site mutant of WaLP, M190A α-lytic protease (MaLP) enzymes [4]. All reagents used were dissolved in 50 mM ammonium bicarbonate. Approximately 20 µg of protein was taken and diluted to 0.25 µg/µL in 50 mM ammonium bicarbonate. Proteins were then reduced (2.5 µL of 50 mM DTT, 56 °C, 30 minutes) and alkylated (110 mM iodoacetamide, 2.5 µL, room temperature, in the dark, 20 minutes). The enzymatic digestion (0.5 µL of a 1 µg/µL enzyme solution) was carried out overnight at 37 °C.

**Sample preparation – trypsin digestion of gel bands**

The marked bands or regions of the lane were cut from the gel with a clean scalpel.  Gel pieces were cut into 1 mm cubes for preparation prior to in-gel digestion. Reagents were prepared in 100 mM ammonium bicarbonate.  The gel pieces were first washed with water, then shrunk with acetonitrile prior to reduction using DTT (56 °C, 30 minutes, 10 mM).  The gel pieces were then shrunk again with acetonitrile and alkylated with iodoacetamide (room temperature, in the dark, 20 minutes, 55 mM).  After shrinking again with acetonitrile, the samples were placed on ice and a volume (sufficient to cover the gel pieces) of trypsin solution (1 ng/µL solution in 50 mM ammonium bicarbonate) was added.  The gel pieces were allowed to swell on ice for 30 minutes, after which they were placed in a shaker overnight at 37 °C for digestion to take place. Peptides were extracted after sonication for 15 minutes, centrifugation and removal of the supernatant. A solution of 50:50 water:acetonitrile, 1% formic acid (2 x the volume of the gel pieces) was added to the mixture in a fresh tube and the samples were again sonicated for 15 minutes and centrifuged where after the supernatant was pooled with the first extract.  The pooled supernatants were dried down with the speed vacuum centrifuge.  The samples were further dissolved in 10 µL of reconstitution buffer (96:4 water:acetonitrile, 0.1% formic acid and analysed by LC-MS/MS.

**LC-MS/MS**

Peptides were separated using the nanoAcquity UPLC system (Waters) fitted with a trapping (nanoAcquity Symmetry C_18_, 5µm, 180 µm x 20 mm) and an analytical column (nanoAcquity BEH C_18_, 1.7µm, 75µm x 200mm). The outlet of the analytical column was coupled directly to an LTQ Orbitrap Velos Pro (Fisher Scientific GmbH, Schwerte, Germany) using the Proxeon nanospray source. Solvent A was water, 0.1 % formic acid and solvent B was acetonitrile, 0.1 % formic acid. The samples (100 fmol) were loaded with a constant flow of solvent A at 5 µL/min onto the trapping column. Trapping time was 6 minutes. Peptides were eluted via the analytical column at a constant flow of 0.3 µL/min. During the elution step, the percentage of solvent B increased in a linear fashion from 3 % to 40 % in 15 minutes. The peptides were introduced into the mass spectrometer via a Pico-Tip Emitter 360 µm OD x 20 µm ID; 10 µm tip (New Objective, Inc., Woburn, USA) and a spray voltage of 2.2 kV was applied. The capillary temperature was set at 300 °C. Full scan MS spectra with mass range 300-1700 *m/z* were acquired in profile mode in the FT with resolution of 30000. The filling time was set at maximum of 500 ms with limitation of 10^6^ ions. The most intense ions (up to 15) from the full scan MS were selected for fragmentation in the LTQ. Normalized collision energy of 40 % was used, and the fragmentation was performed after accumulation of 3 x 10^4^ ions or after filling time of 100 ms for each precursor ion (whichever occurred first). MS/MS data were acquired in centroid mode. Only multiply charged (2+, 3+, 4+) precursor ions were selected for MS/MS. The dynamic exclusion list was restricted to 500 entries with maximum retention period of 30 s and relative mass window of 10 ppm. In order to improve the mass accuracy, a lock mass correction using a background ion (*m/z* 445.12003) was applied.

**Data analysis**

MSConvert (Proteowizard) was used for creating .mgf files from the raw data, needed for searching in MASCOT version 2.2.07 (Matrix Science Ltd, London, UK). The data were searched against the NCBInr (bacteria) database or an in-house user database to which the expected sequences had been appended. Both databases contain a list of common contaminants. The data were searched with the following modifications: Carbamidomethyl (C) (Fixed) and Oxidation (M) (Variable). The mass error tolerance for the full scan MS spectra was set at 20 ppm and for the MS/MS spectra at 0.5 Da. Data were searched with one missed cleavage for tryptic digest data. For WaLP and MaLP enzymes, no enzyme specificity was selected with 0 missed cleavages. Results were processed using Proteinscape 2.1 (Bruker Daltonik GmbH, Bremen, Germany). Mascot score cut-offs of 20 (peptide) and 40 (protein) and p-values < 0.05 were used.

In order to find potential unknown PTMs, an error tolerant search (containing standard Unimod modifications) in Mascot was performed on the data from the identified protein in each of the UserDB searches. Potential PTMs indicated in this search were then added as variable modifications to a repeat search of the UserDB, in addition to the PTMs indicated above.

**Table S1** Plasmids and oligonucleotides used for cloning protein encoding genes and for recombinant protein expression.

| **Protein** | **Gene** | **Origin^a)^** | **Cloning strategy** | **Vector^b)^** | **PCR primer 1 (F) and primer 2 (R)** |
| --- | --- | --- | --- | --- | --- |
| Acr_opt_ | Rv2031c | Synthetic | *Nco*I-*Hin*dIII ligation | pMyNT |  |
| Ag85a_opt_ | Rv3804c | Synthetic | *Nco*I-*Hin*dIII ligation | pMyNT |  |
| Ag85a | Rv3804c | H37Rv | PCR (*Nco*I-*Bam*HI)  *Nco*I-*Bam*HI ligation | pMyC | F 5’-ATACCATGGGCCAGCTTGTTGACAGGGTTCGTG GCG-3’  R 5’-ATAGGATCCGGCGCCCTGGGGCGC-3’ |
| Ag85a^Δ44^ | Rv3804c | H37Rv | PCR (*Nco*I-*Bam*HI)  *Nco*I-*Bam*HI ligation | pMyNT | F 5’-ATACCATGGGCTCCCGGCCGGGCTTGCCGGT-3’  R 5’-ATAGGATCCTCAGGCGCCCTGGGGCGC-3’ |
| Ag85b | Rv1886c | H37Rv | PCR (*Nco*I-*Hin*dIII)  *Nco*I-*Hin*dIII ligation | pMyC | F 5’-ATACCATGGGCACAGACGTGAGCCGAAAGATTC GAGC-3’  R 5’-ATAAAGCTTGGCCGGCGCCTAACGAAC-3’ |
| Ag85b^Δ41^ | Rv1886c | H37Rv | PCR (*Nco*I-*Hin*dIII)  *Nco*I-*Hin*dIII ligation | pMyNT | F 5’-ATACCATGGGCTCCCGGCCGGGGCTGCCGGT-3’  R 5’-ATAAAGCTTTCAGCCGGCGCCTAACGAAC-3’ |
| Apa_opt_ | Rv1860 | Synthetic | *Nco*I-*Hin*dIII ligation | pMyNT |  |
| $\text{Apa}_{\text{opt}}^{\boldsymbol{\Delta}39}$ | Rv1860 | Synthetic | PCR (*Nco*I-*Hin*dIII)  *Nco*I-*Hin*dIII ligation | pMyNT | F 5’-ATACCATGGACCCCGAGCCGGCACCG-3’  R 5’-ATAAAGCTTTCAGGCGGGGAGCG-3’ |
| BfrB | Rv3841 | H37Rv | PCR (*Nco*I-*Hin*dIII)  *Nco*I-*Hin*dIII ligation | pMyNT | F 5’-ATACCATGGGCACAGAATACGAAGGGCCTAAG-3’  R 5’-ATAAAGCTTTCAGAGGCGGCCCCCGG-3’ |
| EchA3 | Rv0632c | H37Rv | PCR (*Pci*I-B*am*HI)  *Nco*I-*Bam*HI ligation | pMyNT | F 5’-ATAACATGTCGAGCGACCCGGTCAGCTATAC-3’  R 5’-ATAGGATCCTCACAGCCCGAACTCGGCTGCTATC-3’ |
| EspA_opt_ | Rv3616c | Synthetic | *Nco*I-*Hin*dIII ligation | pMyNT |  |
| EspB_opt_ | Rv3881c | Synthetic | *Nco*I-*Hin*dIII ligation | pMyNT |  |
| EspC_opt_ | Rv3615c | Synthetic | *Nco*I-*Hin*dIII ligation | pMyNT |  |
| EspE_opt_ | Rv3864 | Synthetic | *Nco*I-*Hin*dIII ligation | pMyNT |  |
| EsxBA | Rv3874/rv3875 | H37Rv | [2] | pMyNT |  |
| GlcB | Rv1837c | H37Rv | PCR (*Nco*I-*Hin*dIII)  *Nco*I-*Hin*dIII ligation | pMyNT | F 5’-ATACCATGGGCACAGATCGCGTGTCGGTGGGC-3’  R 5’-ATAAAGCTTTCAGCGGGCCGCATCGTCA-3’ |
| LprG_opt_ | Rv1411c | Synthetic | *Nco*I-*Hin*dIII ligation | pMyNT |  |
| Mpt64_opt_ | Rv1980 | Synthetic | *Nco*I-*Hin*dIII ligation | pMyNT |  |
| $\text{Mpt64}_{\text{opt}}^{\boldsymbol{\Delta}24}$ | Rv1980 | Synthetic | PCR (*Nco*I-*Hin*dIII)  *Nco*I-*Hin*dIII ligation | pMyNT | F 5’-ATACCATGGCCCCCAAGACCTACTGCGAG-3’  R 5’-ATAAAGCTTTCACGCGAGCATGG-3’ |
| Mpt83_opt_ | Rv2873 | Synthetic | *Nco*I-*Hin*dIII ligation | pMyNT |  |
| $\text{Mpt83}_{\text{opt}}^{\boldsymbol{\Delta}24}$ | Rv2873 | Synthetic | PCR (*Nco*I-*Hin*dIII)  *Nco*I-*Hin*dIII ligation | pMyNT | F 5’-ATACCATGGGCTGCTCCTCCACCAAGCC-3’  R 5’-ATAAAGCTTTCACTGGGCTGGGGGC-3’ |
| PstS1_opt_ | Rv0934 | Synthetic | *Nco*I-*Hin*dIII ligation | pMyNT |  |
| PstS1 | Rv0934 | H37Rv | PCR (*Nco*I-*Hind*III)  *Nco*I-*Hin*dIII ligation | pMyC | F 5’-ATACCATGGGCAAAATTCGTTTGCATACGCTGTTG GCCG-3’  R 5’-ATAAAGCTTGGCTGGAAATCGTCGCGATC-3’ |
| PstS1^Δ24^ | Rv0934 | H37Rv | PCR (*Nco*I-*Hin*dIII)  *Nco*I-*Hin*dIII ligation | pMyNT | F 5’-ATACCATGGGCTCGAAACCACCGAGCGGTTC-3’  R 5’-ATAAAGCTTTCAGCTGGAAATCGTCGCGATC-3’ |
| Rv2654c_opt_ | Rv2654 | Synthetic | *Nco*I-*Hin*dIII ligation | pMyNT |  |

^a)^ Target genes were derived from genomic DNA of *M. tuberculosis* H37Rv or from pUC57 constructs encoding codon-optimized genes synthesized by Genscript as indicated by the “opt” subscript in protein name.

^b)^ Cloning of target genes in pMyNT resulted in fusion proteins with a TEV-cleavable N-terminal His_6_-tag, whereas pMyC constructs encode a non-cleavable His_6_-tag at the C-terminus.

**Table S2** Purification strategy and protein yields after large scale expression of selected proteins

| **Protein** | **Rv number** | **Purification strategy** | **Yield (mg/L)** |
| --- | --- | --- | --- |
| Acr_opt_ | Rv2031c | IMAC 🡺 IEX 🡺 SEC | 2.1 |
| Ag85a^Δ44^ | Rv3804c | IMAC 🡺 IEX 🡺 SEC | 19.4 |
| Ag85b^Δ41^  ^Δ39^ | Rv1886c | IMAC 🡺 IEX 🡺 SEC | 5.8 |
| Apa_opt_ | Rv1860 | IMAC 🡺 SEC | 1.3 |
| **BfrB** | Rv3841 | IMAC 🡺 TEV cleavage 🡺 IMAC subtraction 🡺IEX 🡺 SEC | 0.2 |
| **EchA3** | Rv0632c | IMAC 🡺 SEC | 1.6 |
| **EspB_opt_** | Rv3881c | IMAC 🡺 IEX 🡺 SEC (peak A/B)^a)^ | 0.2/0.6 |
| **EsxBA** | Rv3874/ Rv3875 | IMAC 🡺 IEX 🡺 SEC | 2.7 |
| **GlcB** | Rv1837c | IMAC 🡺 IEX 🡺 HIC 🡺SEC | 0.2 |
| **LprG_opt_**  _opt_ | Rv1411c | IMAC 🡺 SEC | 0.1 |
| **Mpt64^Δ24^** | Rv1980c | IMAC 🡺 IEX 🡺 SEC | 0.3 |
| **PstS1^Δ24^** | Rv0934 | IMAC 🡺 IEX 🡺 SEC | 1.6 |
| **Rv2654c_opt_** | **Rv2654c** | IMAC^b)^ | 0.2 |

^a)^ The elution profile of EspB_opt_ on IEX indicated the presence of two distinct species (denoted as peak A and peak B) eluting at different retention volumes.

^b)^ Antitoxin Rv2654c was not purified by SEC given that the protein precipitated after buffer exchange to SEC buffer and removal of imidazole. The observed protein instability is most likely as a result of co-purification of DNA as deduced from the unusually high A_260_ to A_280_ ratio.

**Table S3** Intact mass measurements by LC-MS

| **Protein** | **Predicted MW (Da)** | **Experimental MW (Da)** |
| --- | --- | --- |
| Acr_opt_ | 18544.8 | 18545 |
| Ag85a^Δ44^ | 33992.0 | 33990 |
| Ag85b^Δ41^  **^Δ39^** | 33020.7 | 33018 |
| **Apa_opt_** | 31228.8 | 31229 |
| **BfrB** | 20627.1 | 20626 |
| **EchA3** | 26759.5 | 26759 |
| **EspB_opt_** (peak A) | 49968.3 | 49966 |
| **EspB_opt_** (peak B) | 49968.3 | 49966 |
| **EsxBA** | 13111.3 (His_6_-EsxB) | 13109 (His_6_-EsxB) |
|  | 9903.8 (EsxA) | ^c)^ |
| **GlcB** | 82777.6 | 82940 |
| **LprG_opt_**  **^Δ24^** | 26922.3 | ^c)^ |
| **Mpt64_opt_** | 24881.8 | 24880 |
| **PstS1^Δ24^** | 38248.7 | 38246 |

^a) b)^ The elution profile of EspB_opt_ on IEX indicated the presence of two distinct species (denoted as peak A and B) eluting at different retention volumes.

^c)^ EsxA and LprG_opt_ were not amenable to intact mass determination, but protein identity was confirmed by follow-up peptide mass fingerprinting analysis (Table S4).

**Table S4** Protein identification analysis of the protein digests (LC-MS/MS) carried out for a subset of proteins

| **Protein** | **Enzyme** | **No. of unique peptide matches** | **% sequence coverage** | **Mascot score**^a)^ | **Second hit**  **(Mascot score)**^b)^ | **Third hit**  **(Mascot score)**^b)^ | **PRIDE name**^c)^ |
| --- | --- | --- | --- | --- | --- | --- | --- |
| **Apa_opt_**  ^Δ39^ | MALP | 113 | 96.7 | 7038 (6416) | gi\|230610^d)^ (2228) | none | …AP041_MALP |
|  | WALP | 132 | 96.7 | 7799 (6755) | gi\|230209^d)^ (6379) | none | …AP041_WALP |
| **EspB_opt_ (peak A)^e)^**  **EspB_opt_ (peak B)^e)^** | Trypsin  Trypsin | 39  37 | 90.4  90.2 | 2425 (2310)  2183 (2037) | gi\|118472423 (684)  gi\|118470561 (361) | gi\|118470561 (242)  gi\|118470265 (99) | …AP5_FIND170_A  …AP5_FIND170_B |
| EsxB (from EsxBA)  EsxA (from EsxBA) | Trypsin  Trypsin | 14  3 | 79  60 | 917 (1024)  270 (301) | gi\|118470265 (368)  EsxB (732)^f)^ | LprG (274)  LprG (299) | …AP3_FIND150upper  …AP4_FIND150lower |
| LprG_opt_ | Trypsin | 17 | 73 | 1108 (908)^g)^ | gi\|118470265 (1303) | gi\|118470248 (969) | …AP1_FIND22 |

^a)^ Mascot score obtained for the expected protein after searching against an in-house database to which the expected sequences (including tags) were appended. The Mascot score obtained for the expected protein after searching against the NCBInr (bacteria) database which included all *M. smegmatis* mc^2^155 and *M. tuberculosis* H37Rv proteins is given between brackets.

^b)^ GenBank identification numbers and Mascot score obtained for the second or third hit after searching against the NCBInr (bacteria) database which included all *M. smegmatis* mc^2^155 and *M. tuberculosis* H37Rv proteins. All secondary and tertiary hits were identified as contaminating proteins from *M. smegmatis* mc^2^155, with the exception of LprG from *M. tuberculosis* H37Rv detected in the EsxBA preparation.

^c)^ The mass spectrometry proteomics data have been deposited to the ProteomeXchange Consortium (<http://proteomecentral.proteomexchange.org>) via the PRIDE partner repository [5] with the dataset identifier PXD004133. The suffix of the filenames used in PRIDE database are abbreviated as shown.

^d)^ Enzyme used for proteolytic digest.

^e)^ The elution profile of EspB_opt_ on IEX indicated the presence of two distinct species (peak A; peak B). Both protein species were purified separately by SEC and analysed individually with LC-MS/MS.

^f)^ EsxB is the first hit after analysis of the EsxA band. This is probably due to contamination from degraded EsxB.

^g)^ The LprG_opt_ sample is significantly contaminated with endogenous proteins derived from the expression strain *M. smegmatis* mc^2^155. In a search against the NCBInr (bacteria) database which included all *M. smegmatis* mc^2^155 and *M. tuberculosis* H37Rv proteins, LprG_opt_ is only the fourth hit with a Mascot score of 908.

**REFERENCES**

[1] Sambrook, J., Fritsch, E. F., Maniatis, T., *Molecular cloning: a laboratory manual*, Cold Spring Harbor Laboratory Press, Cold Spring Harbor 1989.

[2] Poulsen, C., Holton, S., Geerlof, A., Wilmanns, M., Song, Y. H., Stoichiometric protein complex formation and over-expression using the prokaryotic native operon structure. *FEBS letters* 2010, *584*, 669-674.

[3] Snapper, S. B., Melton, R. E., Mustafa, S., Kieser, T., Jacobs, W. R., Jr., Isolation and characterization of efficient plasmid transformation mutants of *Mycobacterium smegmatis*. *Molecular microbiology* 1990, *4*, 1911-1919.

[4] Meyer, J. G., Kim, S., Maltby, D. A., Ghassemian, M.*, et al.*, Expanding proteome coverage with orthogonal-specificity alpha-lytic proteases. *Molecular & cellular proteomics : MCP* 2014, *13*, 823-835.

**Figure S1** Protein sequences

**Acr_opt_**

MK*HHHHHH*PSAGENLYFQGAMATTLPVQRHPRSLFPEFSELFAAFPSFAGLRPTFDTRLMRLEDEMKEGRYEVRAELPGVDPDKDVDIMVRDGQLTIKAERTEQKDFDGRSEFAYGSFVRTVSLPVGADEDDIKATYDKGILTVSVAVSEGKPTEKHIQIRSTN

**Ag85a_opt_**

MK*HHHHHH*PSAGENLYFQGAMGQLVDRVRGAVTGMSRRLVVGAVGAALVSGLVGAVGGTATAGAFSRPGLPVEYLQVPSPSMGRDIKVQFQSGGANSPALYLLDGLRAQDDFSGWDINTPAFEWYDQSGLSVVMPVGGQSSFYSDWYQPACGKAGCQTYKWETFLTSELPGWLQANRHVKPTGSAVVGLSMAASSALTLAIYHPQQFVYAGAMSGLLDPSQAMGPTLIGLAMGDAGGYKASDMWGPKEDPAWQRNDPLLNVGKLIANNTRVWVYCGNGKPSDLGGNNLPAKFLEGFVRTSNIKFQDAYNAGGGHNGVFDFPDSGTHSWEYWGAQLNAMKPDLQRALGATPNTGPAPQGA

**Ag85a**

MGQLVDRVRGAVTGMSRRLVVGAVGAALVSGLVGAVGGTATAGAFSRPGLPVEYLQVPSPSMGRDIKVQFQSGGANSPALYLLDGLRAQDDFSGWDINTPAFEWYDQSGLSVVMPVGGQSSFYSDWYQPACGKAGCQTYKWETFLTSELPGWLQANRHVKPTGSAVVGLSMAASSALTLAIYHPQQFVYAGAMSGLLDPSQAMGPTLIGLAMGDAGGYKASDMWGPKEDPAWQRNDPLLNVGKLIANNTRVWVYCGNGKPSDLGGNNLPAKFLEGFVRTSNIKFQDAYNAGGGHNGVFDFPDSGTHSWEYWGAQLNAMKPDLQRALGATPNTGPAPQGAQAS*HHHHHH*

**Ag85a^Δ44^**

MK*HHHHHH*PSAGENLYFQGAMGSRPGLPVEYLQVPSPSMGRDIKVQFQSGGANSPALYLLDGLRAQDDFSGWDINTPAFEWYDQSGLSVVMPVGGQSSFYSDWYQPACGKAGCQTYKWETFLTSELPGWLQANRHVKPTGSAVVGLSMAASSALTLAIYHPQQFVYAGAMSGLLDPSQAMGPTLIGLAMGDAGGYKASDMWGPKEDPAWQRNDPLLNVGKLIANNTRVWVYCGNGKPSDLGGNNLPAKFLEGFVRTSNIKFQDAYNAGGGHNGVFDFPDSGTHSWEYWGAQLNAMKPDLQRALGATPNTGPAPQGA

**Ag85b**

MGSRPGLPVEYLQVPSPSMGRDIKVQFQSGGNNSPAVYLLDGLRAQDDYNGWDINTPAFEWYYQSGLSIVMPVGGQSSFYSDWYSPACGKAGCQTYKWETFLTSELPQWLSANRAVKPTGSAAIGLSMAGSSAMILAAYHPQQFIYAGSLSALLDPSQGMGPSLIGLAMGDAGGYKAADMWGPSSDPAWERNDPTQQIPKLVANNTRLWVYCGNGTPNELGGANIPAEFLENFVRSSNLKFQDAYNAAGGHNAVFNFPPNGTHSWEYWGAQLNAMKGDLQSSLGAGQAS*HHHHHH*

**Ag85b^Δ41^**

MK*HHHHHH*PSAGENLYFQGAMGSRPGLPVEYLQVPSPSMGRDIKVQFQSGGNNSPAVYLLDGLRAQDDYNGWDINTPAFEWYYQSGLSIVMPVGGQSSFYSDWYSPACGKAGCQTYKWETFLTSELPQWLSANRAVKPTGSAAIGLSMAGSSAMILAAYHPQQFIYAGSLSALLDPSQGMGPSLIGLAMGDAGGYKAADMWGPSSDPAWERNDPTQQIPKLVANNTRLWVYCGNGTPNELGGANIPAEFLENFVRSSNLKFQDAYNAAGGHNAVFNFPPNGTHSWEYWGAQLNAMKGDLQSSLGAG

**Apa_opt_**

MK*HHHHHH*PSAGENLYFQGAMGHQVDPNLTRRKGRLAALAIAAMASASLVTVAVPATANADPEPAPPVPTTAASPPSTAAAPPAPATPVAPPPPAAANTPNAQPGDPNAAPPPADPNAPPPPVIAPNAPQPVRIDNPVGGFSFALPAGWVESDAAHFDYGSALLSKTTGDPPFPGQPPPVANDTRIVLGRLDQKLYASAEATDSKAAARLGSDMGEFYMPYPGTRINQETVSLDANGVSGSASYYEVKFSDPSKPNGQIWTGVIGSPAANAPDAGPPQRWFVVWLGTANNPVDKGAAKALAESIRPLVAPPPAPAPAPAEPAPAPAPAGEVAPTPTTPTPQRTLPA

$$\text{Apa}_{\text{opt}}^{\boldsymbol{\Delta39}}$$

MK*HHHHHH*PSAGENLYFQGAMDPEPAPPVPTTAASPPSTAAAPPAPATPVAPPPPAAANTPNAQPGDPNAAPPPADPNAPPPPVIAPNAPQPVRIDNPVGGFSFALPAGWVESDAAHFDYGSALLSKTTGDPPFPGQPPPVANDTRIVLGRLDQKLYASAEATDSKAAARLGSDMGEFYMPYPGTRINQETVSLDANGVSGSASYYEVKFSDPSKPNGQIWTGVIGSPAANAPDAGPPQRWFVVWLGTANNPVDKGAAKALAESIRPLVAPPPAPAPAPAEPAPAPAPAGEVAPTPTTPTPQRTLPA

**BfrB**

MK*HHHHHH*PSAGENLYFQGAMGTEYEGPKTKFHALMQEQIHNEFTAAQQYVAIAVYFDSEDLPQLAKHFYSQAVEERNHAMMLVQHLLDRDLRVEIPGVDTVRNQFDRPREALALALDQERTVTDQVGRLTAVARDEGDFLGEQFMQWFLQEQIEEVALMATLVRVADRAGANLFELENFVAREVDVAPAASGAPHAAGGRL

**EchA3**

MK*HHHHHH*PSAGENLYFQ*G*AMSSDPVSYTRKDSIAVISMDDGKVNALGPAMQQALNAAIDNADRDDVGALVITGNGRVFSGGFDLKILTSGEVQPAIDMLRGGFELAYRLLSYPKPVVMACTGHAIAMGAFLLSCGDHRVAAHAYNIQANEVAIGMTIPYAALEIMKLRLTRSAYQQATGLAKTFFGETALAAGFIDEIALPEVVVSRAEEAAREFAGLNQHAHAATKLRSRADALTAIRAGIDGIAAEFGL

**EspA_opt_**

MK*HHHHHH*PSAGENLYFQGAMGSRAFIIDPTISAIDGLYDLLGIGIPNQGGILYSSLEYFEKALEELAAAFPGDGWLGSAADKYAGKNRNHVNFFQELADLDRQLISLIHDQANAVQTTRDILEGAKKGLEFVRPVAVDLTYIPVVGHALSAAFQAPFCAGAMAVVGGALAYLVVKTLINATQLLKLLAKLAELVAAAIADIISDVADIIKGTLGEVWEFITNALNGLKELWDKLTGWVTGLFSRGWSNLESFFAGVPGLTGATSGLSQVTGLFGAAGLSASSGLAHADSLASSASLPALAGIGGGSGFGGLPSLAQVHAASTRQALRPRADGPVGAAAEQVGGQSQLVSAQGSQGMGGPVGMGGMHPSSGASKGTTTKKYSEGAAAGTEDAERAPVEADAGGGQKVLVRNVV

**EspB_opt_**

MK*HHHHHH*PSAGENLYFQGAMGTQSQTVTVDQQEILNRANEVEAPMADPPTDVPITPCELTAAKNAAQQLVLSADNMREYLAAGAKERQRLATSLRNAAKAYGEVDEEAATALDNDGEGTVQAESAGAVGGDSSAELTDTPRVATAGEPNFMDLKEAARKLETGDQGASLAHFADGWNTFNLTLQGDVKRFRGFDNWEGDAATACEASLDQQRQWILHMAKLSAAMAKQAQYVAQLHVWARREHPTYEDIVGLERLYAENPSARDQILPVYAEYQQRSEKVLTEYNNKAALEPVNPPKPPPAIKIDPPPPPQEQGLIPGFLMPPSDGSGVTPGTGMPAAPMVPPTGSPGGGLPADTAAQLTSAGREAAALSGDVAVKAASLGGGGGGGVPSAPLGSAIGGAESVRPAGAGDIAGLGQGRAGGGAALGGGGMGMPMGAAHQGQGGAKSKGSQQEDEALYTEDRAWTEAVIGNRRRQDSKESK

**EspC_opt_**

MK*HHHHHH*PSAGENLYFQGAMGTENLTVQPERLGVLASHHDNAAVDASSGVEAAAGLGESVAITHGPYCSQFNDTLNVYLTAHNALGSSLHTAGVDLAKSLRIAAKIYSEADEAWRKAIDGLFT

**EspE_opt_**

MK*HHHHHH*PSAGENLYFQGAMASGSGLCKTTSNFIWGQLLLLGEGIPDPGDIFNTGSSLFKQISDKMGLAIPGTNWIGQAAEAYLNQNIAQQLRAQVMGDLDKLTGNMISNQAKYVSDTRDVLRAMKKMIDGVYKVCKGLEKIPLLGHLWSWELAIPMSGIAMAVVGGALLYLTIMTLMNATNLRGILGRLIEMLTTLPKFPGLPGLPSLPDIIDGLWPPKLPDIPIPGLPDIPGLPDFKWPPTPGSPLFPDLPSFPGFPGFPEFPAIPGFPALPGLPSIPNLFPGLPGLGDLLPGVGDLGKLPTWTELAALPDFLGGFAGLPSLGFGNLLSFASLPTVGQVTATMGQLQQLVAAGGGPSQLASMGSQQAQLISSQAQQGGQQHATLVSDKKEDEEGVAEAERAPIDAGTAASQRGQEGTVL

**EsxBA**

MK*HHHHHH*PSAGENLYFQGAMAEMKTDAATLAQEAGNFERISGDLKTQIDQVESTAGSLQGQWRGAAGTAAQAAVVRFQEAANKQKQELDEISTNIRQAGVQYSRADEEQQQALSSQMGF (EsxB)

MTEQQWNFAGIEAAASAIQGNVTSIHSLLDEGKQSLTKLAAAWGGSGSEAYQGVQQKWDATATELNNALQNLARTISEAGQAMASTEGNVTGMFA (EsxA)

**GlcB**

MK*HHHHHH*PSAGENLYFQGAMGTDRVSVGNLRIARVLYDFVNNEALPGTDIDPDSFWAGVDKVVADLTPQNQALLNARDELQAQIDKWHRRRVIEPIDMDAYRQFLTEIGYLLPEPDDFTITTSGVDAEITTTAGPQLVVPVLNARFALNAANARWGSLYDALYGTDVIPETDGAEKGPTYNKVRGDKVIAYARKFLDDSVPLSSGSFGDATGFTVQDGQLVVALPDKSTGLANPGQFAGYTGAAESPTSVLLINHGLHIEILIDPESQVGTTDRAGVKDVILESAITTIMDFEDSVAAVDAADKVLGYRNWLGLNKGDLAAAVDKDGTAFLRVLNRDRNYTAPGGGQFTLPGRSLMFVRNVGHLMTNDAIVDTDGSEVFEGIMDALFTGLIAIHGLKASDVNGPLINSRTGSIYIVKPKMHGPAEVAFTCELFSRVEDVLGLPQNTMKIGIMDEERRTTVNLKACIKAAADRVVFINTGFLDRTGDEIHTSMEAGPMVRKGTMKSQPWILAYEDHNVDAGLAAGFSGRAQVGKGMWTMTELMADMVETKIAQPRAGASTAWVPSPTAATLHALHYHQVDVAAVQQGLAGKRRATIEQLLTIPLAKELAWAPDEIREEVDNNCQSILGYVVRWVDQGVGCSKVPDIHDVALMEDRATLRISSQLLANWLRHGVITSADVRASLERMAPLVDRQNAGDVAYRPMAPNFDDSIAFLAAQELILSGAQQPNGYTEPILHRRRREFKARAAEKPAPSDRAGDDAAR

**LprG_opt_**

MK*HHHHHH*PSAGENLYFQGAMGRTPRRHCRRIAVLAAVSIAATVVAGCSSGSKPSGGPLPDAKPLVEEATAQTKALKSAHMVLTVNGKIPGLSLKTLSGDLTTNPTAATGNVKLTLGGSDIDADFVVFDGILYATLTPNQWSDFGPAADIYDPAQVLNPDTGLANVLANFADAKAEGRDTINGQNTIRISGKVSAQAVNQIAPPFNATQPVPATVWIQETGDHQLAQAQLDRGSGNSVQMTLSKWGEKVQVTKPPVS

**Mpt64_opt_**

MK*HHHHHH*PSAGENLYFQGAMGRIKIFMLVTAVVLLCCSGVATAAPKTYCEELKGTDTGQACQIQMSDPAYNINISLPSYYPDQKSLENYIAQTRDKFLSAATSSTPREAPYELNITSATYQSAIPPRGTQAVVLKVYQNAGGTHPTTTYKAFDWDQAYRKPITYDTLWQADTDPLPVVFPIVQGELSKQTGQQVSIAPNAGLDPVNYQNFAVTNDGVIFFFNPGELLPEAAGPTQVLVPRSAIDSMLA

$$\text{Mpt64}_{\text{opt}}^{\boldsymbol{\Delta24}}$$

MK*HHHHHH*PSAGENLYFQGAMAPKTYCEELKGTDTGQACQIQMSDPAYNINISLPSYYPDQKSLENYIAQTRDKFLSAATSSTPREAPYELNITSATYQSAIPPRGTQAVVLKVYQNAGGTHPTTTYKAFDWDQAYRKPITYDTLWQADTDPLPVVFPIVQGELSKQTGQQVSIAPNAGLDPVNYQNFAVTNDGVIFFFNPGELLPEAAGPTQVLVPRSAIDSMLA

**Mpt83_opt_**

MK*HHHHHH*PSAGENLYFQGAMGINVQAKPAAAASLAAIAIAFLAGCSSTKPVSQDTSPKPATSPAAPVTTAAMADPAADLIGRGCAQYAAQNPTGPGSVAGMAQDPVATAASNNPMLSTLTSALSGKLNPDVNLVDTLNGGEYTVFAPTNAAFDKLPAATIDQLKTDAKLLSSILTYHVIAGQASPSRIDGTHQTLQGADLTVIGARDDLMVNNAGLVCGGVHTANATVYMIDTVLMPPAQ

$$\text{Mpt83}_{\text{opt}}^{\boldsymbol{\Delta24}}$$

MK*HHHHHH*PSAGENLYFQGAMGCSSTKPVSQDTSPKPATSPAAPVTTAAMADPAADLIGRGCAQYAAQNPTGPGSVAGMAQDPVATAASNNPMLSTLTSALSGKLNPDVNLVDTLNGGEYTVFAPTNAAFDKLPAATIDQLKTDAKLLSSILTYHVIAGQASPSRIDGTHQTLQGADLTVIGARDDLMVNNAGLVCGGVHTANATVYMIDTVLMPPAQ

**PstS1_opt_**

MK*HHHHHH*PSAGENLYFQGAMGKIRLHTLLAVLTAAPLLLAAAGCGSKPPSGSPETGAGAGTVATTPASSPVTLAETGSTLLYPLFNLWGPAFHERYPNVTITAQGTGSGAGIAQAAAGTVNIGASDAYLSEGDMAAHKGLMNIALAISAQQVNYNLPGVSEHLKLNGKVLAAMYQGTIKTWDDPQIAALNPGVNLPGTAVVPLHRSDGSGDTFLFTQYLSKQDPEGWGKSPGFGTTVDFPAVPGALGENGNGGMVTGCAETPGCVAYIGISFLDQASQRGLGEAQLGNSSGNFLLPDAQSIQAAAAGFASKTPANQAISMIDGPAPDGYPIINYEYAIVNNRQKDAATAQTLQAFLHWAITDGNKASFLDQVHFQPLPPAVVKLSDALIATISS

**PstS1**

MGKIRLHTLLAVLTAAPLLLAAAGCGSKPPSGSPETGAGAGTVATTPASSPVTLAETGSTLLYPLFNLWGPAFHERYPNVTITAQGTGSGAGIAQAAAGTVNIGASDAYLSEGDMAAHKGLMNIALAISAQQVNYNLPGVSEHLKLNGKVLAAMYQGTIKTWDDPQIAALNPGVNLPGTAVVPLHRSDGSGDTFLFTQYLSKQDPEGWGKSPGFGTTVDFPAVPGALGENGNGGMVTGCAETPGCVAYIGISFLDQASQRGLGEAQLGNSSGNFLLPDAQSIQAAAAGFASKTPANQAISMIDGPAPDGYPIINYEYAIVNNRQKDAATAQTLQAFLHWAITDGNKASFLDQVHFQPLPPAVVKLSDALIATISSQAS*HHHHHH*

**PstS1^Δ24^**

MK*HHHHHH*PSAGENLYFQGAMGSKPPSGSPETGAGAGTVATTPASSPVTLAETGSTLLYPLFNLWGPAFHERYPNVTITAQGTGSGAGIAQAAAGTVNIGASDAYLSEGDMAAHKGLMNIALAISAQQVNYNLPGVSEHLKLNGKVLAAMYQGTIKTWDDPQIAALNPGVNLPGTAVVPLHRSDGSGDTFLFTQYLSKQDPEGWGKSPGFGTTVDFPAVPGALGENGNGGMVTGCAETPGCVAYIGISFLDQASQRGLGEAQLGNSSGNFLLPDAQSIQAAAAGFASKTPANQAISMIDGPAPDGYPIINYEYAIVNNRQKDAATAQTLQAFLHWAITDGNKASFLDQVHFQPLPPAVVKLSDALIATISS

**Rv2654c_opt_**

MK*HHHHHH*PSAGENLYFQGAMGSGHALAARTLLAAADELVGGPPVEASAAALAGDAAGAWRTAAVELARALVRAVAESHGVAAVLFAATAAAAAAVDRGDPP

**Legend Figure S1** Amino acid sequences of the different recombinant proteins as produced in *M. smegmatis* upon cloning in pMyNT or pMyC plasmid. His_6_-tag and TEV cleavage site (ENLYFQG) (both arising from the cloning procedure) are in italics and underlined, respectively.

**Figure S2** Size exclusion chromatography analysis of recombinant *M. tuberculosis* proteins tested in multiplex immunoassays

**Acr_opt_**

B

A

**Ag85a^Δ44^**

B

A

**Ag85b^Δ41^**

A

B

**Apa^Δ39^**

**_opt_**

B

A

**BfrB**

B

A

**EchA3**

B

A

**EspB_opt_ peak A**

B

A

**EspB_opt_ peak B**

**__**

A

B

**EsxBA**

B

A

**GlcB**

B

A

**LprG_opt_**

B

A

**Mpt64^Δ24^**

**_opt_**

A

B

**PstS1^Δ24^**

B

A

**Legend Figure S2** (A) SEC chromatograms showing the elution profile and peak elution volume of proteins injected onto a HiLoad Superdex 200 (Acr_opt_, Ag85b^Δ41^, EchA3, EspB_opt_, EsxBA, GlcB, LprGopt, Mpt64_opt_^Δ24^, PstS1^Δ24^) or HiLoad Superdex 75 (Ag85a^Δ44^, Apa_opt_^Δ39^, BfrB) 16/60 prep grade column (GE Healthcare). Composition of the SEC running buffers are described in supplementary Materials and Methods. Peak fractions indicated by the arrow were subsequently analysed by SDS-PAGE. All columns were packed in our laboratory and calibrated with a mixture of three proteins, bovine γ-globulin (IGG; 158 kDa), chicken ovalbumin (Ova; 44 kDa) and equine myoglobin (Myo; 17 kDa) (as part of Gel Filtration Standard mixture, Bio-Rad). The inset shows the corresponding calibration curve and derived linear equation between the logarithm of the molecular weight of the protein in Dalton (log MW) and the retention volume (Rt) in mL. White/open diamond symbols correspond to the theoretical molecular weight of the protein (MW_theoretical_; see Fig. S1). The log MW of each protein was estimated by comparison with the standard proteins and is depicted with a coloured diamond symbol in the inset: blue (for MW_calculated_ > MW_theoretical_), green (for MW_calculated_ < MW_theoretical_), or pink (for MW_calculated_ = MW_theoretical_). (B) Corresponding peak fractions from SEC resolved on a 4-12% Bis-Tris NuPAGE gel and stained with Instant Blue gel stain. Protein size was verified by comparison with the Rotimark protein marker. Protein fractions containing pure protein (indicated by a box) were pooled and concentrated (see Figure S3).

**Figure S3** Protein purity of final preparations analysed by multiplex immunoassays

**Legend Figure S3** SDS-PAGE analysis of final protein preparations polishing with SEC. 2-5 μg of protein was resolved on a 4-12% Bis-Tris NuPAGE gel and stained with Instant Blue gel stain. Protein size was verified by comparison with the Rotimark protein marker.

REFERENCES

[1] Sambrook, J., Fritsch, E. F., Maniatis, T., *Molecular cloning: a laboratory manual*, Cold Spring Harbor Laboratory Press, Cold Spring Harbor 1989.

[2] Poulsen, C., Holton, S., Geerlof, A., Wilmanns, M., Song, Y. H., Stoichiometric protein complex formation and over-expression using the prokaryotic native operon structure. *FEBS letters* 2010, *584*, 669-674.

[3] Snapper, S. B., Melton, R. E., Mustafa, S., Kieser, T., Jacobs, W. R., Jr., Isolation and characterization of efficient plasmid transformation mutants of *Mycobacterium smegmatis*. *Molecular microbiology* 1990, *4*, 1911-1919.

[4] Meyer, J. G., Kim, S., Maltby, D. A., Ghassemian, M.*, et al.*, Expanding proteome coverage with orthogonal-specificity alpha-lytic proteases. *Molecular & cellular proteomics : MCP* 2014, *13*, 823-835.

[5] Vizcaino, J. A., Csordas, A., del-Toro, N., Dianes, J. A.*, et al.*, 2016 update of the PRIDE database and its related tools. *Nucleic acids research* 2016, *44*, D447-456.
